# I looked at you, you looked at me, I smiled at you, you smiled at me—The impact of eye contact on emotional mimicry

**DOI:** 10.3389/fpsyg.2022.970954

**Published:** 2022-09-29

**Authors:** Heidi Mauersberger, Till Kastendieck, Ursula Hess

**Affiliations:** Department of Psychology, Humboldt-Universität zu Berlin, Berlin, Germany

**Keywords:** emotional mimicry, eye contact, affiliation, top-down modulation, interpersonal closeness

## Abstract

Eye contact is an essential element of human interaction and direct eye gaze has been shown to have effects on a range of attentional and cognitive processes. Specifically, direct eye contact evokes a positive affective reaction. As such, it has been proposed that obstructed eye contact reduces emotional mimicry (i.e., the imitation of our counterpart’s emotions). So far, emotional mimicry research has used averted-gaze faces or unnaturally covered eyes (with black censor bars) to analyze the effect of eye contact on emotional mimicry. However, averted gaze can also signal disinterest/ disengagement and censor bars obscure eye-adjacent areas as well and hence impede emotion recognition. In the present study (*N* = 44), we used a more ecological valid approach by showing photos of actors who expressed either happiness, sadness, anger, or disgust while either wearing mirroring sunglasses that obstruct eye contact or clear glasses. The glasses covered only the direct eye region but not the brows, nose ridge, and cheeks. Our results confirm that participants were equally accurate in recognizing the emotions of their counterparts in both conditions (sunglasses vs. glasses). Further, in line with our hypotheses, participants felt closer to the targets and mimicked affiliative emotions more intensely when their counterparts wore glasses instead of sunglasses. For antagonistic emotions, we found the opposite pattern: Disgust mimicry, which was interpreted as an affective reaction rather than genuine mimicry, could be only found in the sunglasses condition. It may be that obstructed eye contact increased the negative impression of disgusted facial expressions and hence the negative feelings disgust faces evoked. The present study provides further evidence for the notion that eye contact is an important prerequisite for emotional mimicry and hence for smooth and satisfying social interactions.

## Introduction

Eye contact is typically part of face-to-face conversations, be it if we talk to our neighbor in the morning on the doorstep or if we chat with our best friend during a cup of tea in the afternoon. Eye contact is an important social signal of attention and affection—not only within human conversations but also across mammals (Emery, 2000). Typically, our eyes meet our counterpart’s eyes several times during a social exchange and the frequency of mutual eye contact goes along with a variety of processes that foster social interaction quality such as likability, attractiveness, positive emotions, approach motivation, and affiliation (Leary, 1957) as the fundamental motive to connect with others (e.g., Mason et al., 2005; Hietanen et al., 2018; Kompatsiari et al., 2019; Niedźwiecka, 2020). A recent study with the closest living non-human primates even suggests that differences in social behaviors between bonobos and chimpanzees such as more cooperative and affiliative behaviors and less acts of aggression in bonobos compared to chimpanzees may be partly explained by the fact that bonobos engage in more frequent and longer mutual eye gaze (Mulholland et al., 2020).

This suggests that when eye contact is obstructed, the affiliative bond between interaction partners is impaired. Just imagine how it feels if you talked to someone who does not look into your eyes during the entire length of the conversation—not even a single time. Or maybe their eyes meet your gaze very shortly a few times but then shift back to the side or to some indistinct point behind you. Would you not feel disliked and would you not try to end the conversation soon rather than later? Indeed, obstructed eye contact creates feelings of unpleasantness, it impedes liking and decreases positive affective reactions to social interaction partners (e.g., Schmitz et al., 2012; Hietanen, 2018; Hietanen et al., 2020) —the latter is also true if a person sees others’ eyes but believes others cannot see theirs (Hietanen and Peltola, 2021). Further, obstructed eye contact may hinder processes that help to regulate and navigate social interactions by establishing and reinforcing social connectedness. One of these fundamental processes that smoothens social interactions and fosters affiliation is emotional mimicry.

### Eye contact and emotional mimicry

Emotional mimicry (i.e., the imitation of our counterpart’s emotional expression) is an automatic but goal-dependent process that both fosters and depends on an affiliative link between mimicker and mimickee (e.g., Hess and Fischer, 2013; Fischer and Hess, 2017; Hess, 2021). That is, successful social interactions where interaction partners feel understood, liked, and cared about require emotional mimicry. Yet, emotional mimicry is very sensitive to contextual influences. Even though it is considered to be an automatic process (Dimberg et al., 2002), research and recent theories have pointed to strong top-down influences on this process (Fischer and Hess, 2017; Kastendieck et al., 2020; Hess, 2021). Specifically, an affiliative stance is a crucial prerequisite for emotional mimicry. In contrast, signs of rejection or disaffiliation reduce or even completely prevent mimicry. In fact, competing opponents, outgroup members, and people who socially exclude us are typically not mimicked and even potentially evoke counter-mimicry (e.g., Lanzetta and Englis, 1989; Bourgeois and Hess, 2008; Likowski et al., 2008; Weyers et al., 2009; van der Schalk et al., 2011; Hühnel et al., 2018). Accordingly, if obstructed eye contact decreases the affiliative link between interaction partners, it should impair emotional mimicry with all its benefits for social exchanges. In line with these considerations, obstructed eye contact has been found to reduce emotional mimicry (e.g., Rychlowska et al., 2012; Imafuku et al., 2020; Kuang et al., 2021).

Yet, this research used unnaturally covered eyes (with black censor bars) or averted-gaze faces to operationalize the lack of eye contact (Rychlowska et al., 2012; Wang and de C Hamilton, 2014; Marschner et al., 2015; Leng et al., 2018; Imafuku et al., 2020; Kuang et al., 2021). Thus, even though these findings point to the importance of eye contact for emotional mimicry research, their results allow for a range of alternative explanations. First, black sensor bars are most often used when people are shown who have been accused of a crime. Thus, black censor bars bear the connotation of moral wrongdoing, which weakens the goal to affiliate with individuals shown with them (Rozin et al., 2008). Second, censor bars obscure eye-adjacent areas and hence may impede emotional understanding, which, in turn, may reduce emotional mimicry. Indeed, several recent studies found that partial face occlusion impairs emotion recognition (Grundmann et al., 2021; McCrackin et al., 2021; Pazhoohi et al., 2021), which then can impact on mimicry (Kastendieck et al., 2021). Further, you are unlikely to meet a person with a black sensor bar in front of their eyes in real life. Hence, these kinds of stimuli clearly lack ecological validity.

Measuring mimicry toward averted-gaze faces is also problematic. It is very likely that the averted gaze may be interpreted as a sign of disinterest, disengagement, rejection, or even social exclusion. Indeed, averted gaze causes individuals to feel ostracized and to devalue the interaction (Wirth et al., 2010; Leng et al., 2018). Consequently, it may be not the obstructed eye contact *as such* but rather the disinterest and rejection averted gaze implies that explains why these stimuli were not mimicked in past studies. Hence, to investigate the influence of eye contact on emotional mimicry, it is necessary to use a paradigm—that (a) does not cover areas of the face relevant for emotion recognition, (b) is ecologically valid and (c) disentangles the presence or absence of eye contact from the deliberate act of looking away from the interaction partner, as the latter combines the absence of eye contact with a clear signal of disinterest. Our study used targets wearing sunglasses (versus clear glasses) to investigate the question whether obstructed eye contact decreases the perceived interpersonal closeness between participants and their interaction partners and hence impairs emotional mimicry of interaction partners.

### Eye contact and emotional mimicry of affiliative versus antagonistic emotions

Emotional displays are social messages that inform about the social intentions of their expressers (Hareli and Hess, 2012). Hence, even without any additional context, they already signal whether or not affiliation and bonding are desired and thus possible. If I smile at you, I seem to be open and willing to get to know you. In contrast, when I sneer and curl the lip, I may seem disgusted by what I think you have done and I may recoil from you. Obviously, only the former of the two expressions communicates affiliative intent and triggers a genuine mimicry response. The latter expression aims to distance oneself from the object or subject of aversion. Thus, if a mimicry response can be observed here, it is very likely that it may not be mimicry *per se*, but rather an *affective* reaction that resembles mimicry, as it leads to the activation of the same facial muscles (Hess, 2021). In this vein, several recent studies could show that antagonistic mimicry (i.e., the mimicry of antagonistic emotions) has the exact opposite effects for social interaction satisfaction than affiliative mimicry (Mauersberger et al., 2015; Mauersberger and Hess, 2019). Thus, antagonistic mimicry leads to feelings of being misunderstood and mutual dislike. Antagonistic mimicry, in turn, may be more likely triggered in a situation where affiliation intentions are impaired due to obstructed eye contact. That is, for antagonistic emotions, we expect the opposite pattern as for affiliative emotions: Sunglasses should increase antagonistic mimicry, whereas they should impair affiliative mimicry.

### The present study

In the present study, we measured mimicry toward actors who expressed either happiness, sadness, anger, or disgust while either wearing mirroring sunglasses that obstructed eye contact or clear glasses. Both types of glasses covered only the direct eye region but not the brows, nose ridge, and cheeks, which are relevant facial areas for distinguishing happiness, sadness, anger, and disgust from each other (Boucher and Ekman, 1975). This is important, as partial occlusion of the face such as wearing face masks may impede emotion recognition (Grundmann et al., 2021; McCrackin et al., 2021; Pazhoohi et al., 2021), and impaired emotion recognition, in turn, may be an alternative explanation in case mimicry is absent or reduced during obstructed eye contact (Kastendieck et al., 2021). Yet, even though face masks reduce emotion recognition rates to a greater extent than sunglasses, sunglasses that have thick bridges may also occlude diagnostic facial areas such as the wrinkles between the brows, which indicate a negative facial expression. Hence, the recognition of emotions such as anger and sadness (and also fear) may be impaired if sunglasses are shaped in a way that they hide the potential wrinkles between the brows (as it was the case in these two studies: Noyes et al., 2021; Kim et al., 2022).

Further, sunglasses merely physically obstruct eye contact; that is, even though targets presumably look at participants, their eyes and hence the direction of their gaze are invisible. This uncertainty may create some kind of interpersonal distance and hence impair emotional mimicry of affiliative emotions. In fact, targets wearing sunglasses were judged to be less trustworthy than targets wearing glasses, presumably because “they render the eyes invisible” and hence destroy the affiliative bond between interaction partners (Graham and Ritchie, 2019). In contrast, this ambiguous (slightly non-affiliative) context produced by obstructed eye contact due to the sunglasses may foster emotional mimicry of antagonistic emotions (i.e., an affective reaction that only resembles emotional mimicry but is not mimicry in the classical sense). Hence, we expected that individuals feel closer to others who wear clear glasses instead of sunglasses and mimic others’ happiness, sadness, and anger^1^ more intensely when they were clear glasses instead of sunglasses. Disgust mimicry, however, should be more pronounced when targets wear sunglasses instead of clear glasses.

## Materials and methods

### Participants

**Figure 1:**
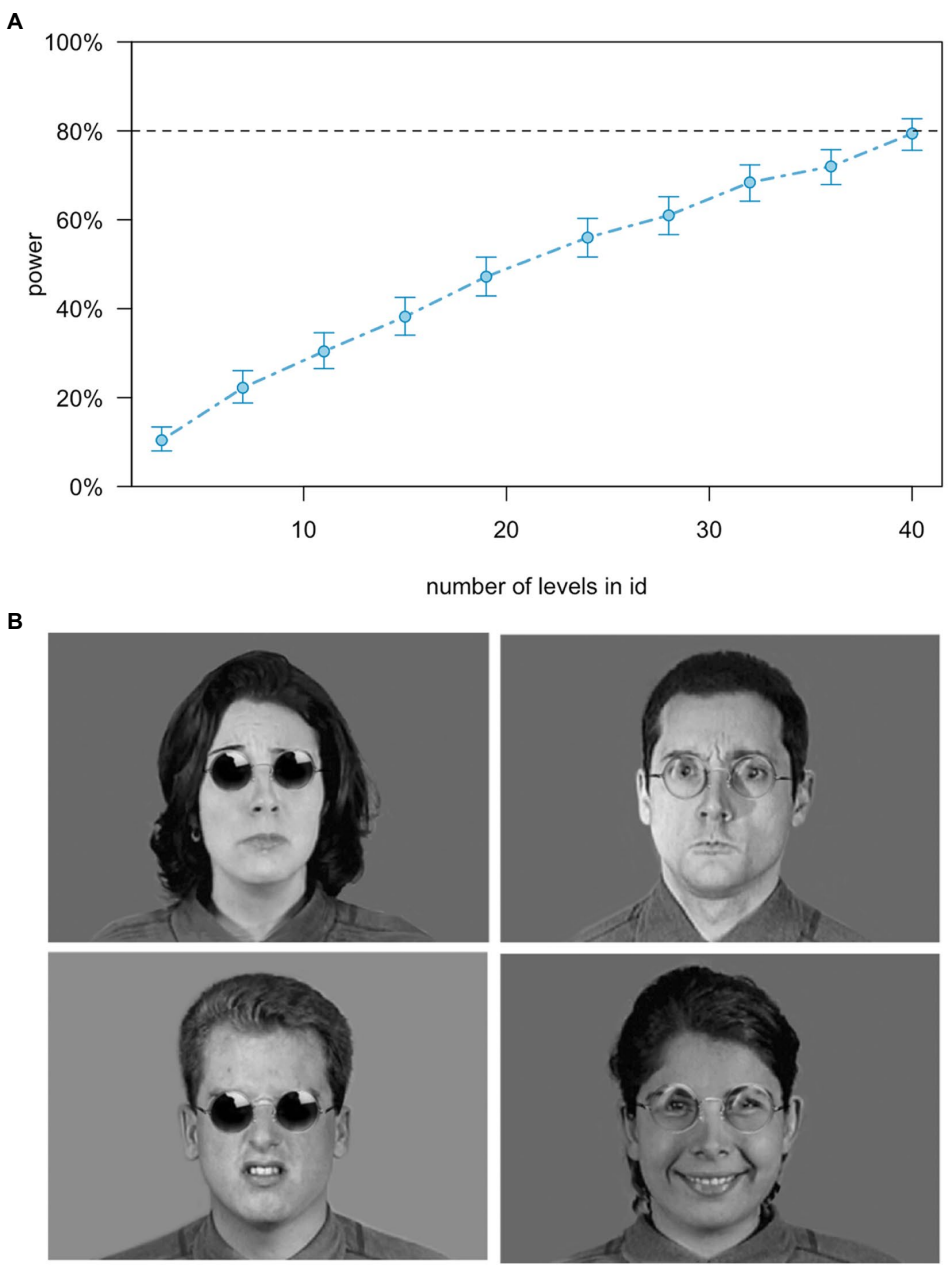
Power curve to estimate the minimum sample size based on data from a pilot study in our laboratory with averted vs. direct gaze **(A)** and example stimuli for each eye contact and each emotion taken from the Montreal Set of Facial Displays of Emotion (Beaupré et al., 2000). Obstructed eye contact (left) and unobstructed eye contact (right), and of sadness (top left), anger (top right), disgust (bottom left), and happiness (bottom right) **(B)**.

Based on a pilot study where we showed participants targets displaying happiness with direct and averted gaze (and where we also measured participants’ emotional mimicry), we used the *simr* package (Green and Macleod, 2016) to run a simulation-based linear mixed model (LMM) power analysis with the *lme4* package (Bates et al., 2014). That is, assuming a similar effect size for emotional mimicry here as in the pilot study, we used the coefficients of the fixed and random effects of an analysis like the planned one to calculate the minimum sample size for achieving at least 80% power at an alpha level of.05. The analysis pointed to recruiting at least 40 participants (see power curve in Figure 1A). Hence, to account for missing EMG data due to technical problems or artifact, we recruited 44 participants (10% oversampling) *via* the Humboldt-Universität zu Berlin’s own recruitment platform PESA (Psychologischer Experimental Server Adlershof), Facebook, and flyers. Hence, our final sample consisted of 44 participants (29 women) with a mean age of 25.3 years (SD = 7.73).

Participants were students or recent students (68% psychology students). All participants were German native speakers. The study was carried out in accordance with the guidelines of the Declaration of Helsinki (except for lack of preregistration) and was approved by the institutional ethics committee. Participants were aware that they had the right to discontinue participation at any time and that their responses were confidential. Participants were informed of the experimental procedure and gave written consent prior to the start of the laboratory session. They participated individually and received either course credit (the 30 psychology students) or a small gift of a value equivalent to €10.

### Stimulus material

The stimulus set we used consisted of black-and-white portrait photographs of four men and four women, taken from the *Montreal Set of Facial Displays of Emotion* (MSFDE; Beaupré et al., 2000) either showing happiness, anger, sadness, disgust, or a neutral face (making 40 photographs in total). All models faced the camera directly with a 0° angle and gaze direction exactly at the observer. To manipulate eye contact in a naturalistic way, we digitally added sunglasses or clear glasses onto the targets’ faces using *Adobe Photoshop* (making 80 stimuli in total). The glasses were round-shaped and did not cover the respective facial muscles involved in the emotional expressions under investigation (see Figure 1B).

Each participant saw two men and two women with glasses displaying all four emotions during the emotion recognition task (plus the neutral expression afterward as manipulation check) and the remaining two men and two women with sunglasses displaying all four emotions during the emotion recognition task (plus the neutral expression afterward as manipulation check). The target-type-of-glasses combinations were fixed for each participant and counterbalanced across participants.

### Procedure

After providing informed consent, participants reclined in a comfortable chair while physiological sensors were attached. Participants sat about 1 m away from the screen, which was a 30″ monitor. The experimenter then left the room, monitored the experiment *via* a video camera, and explained the task *via* microphone. A 3.5-min baseline period for the EMG measures was taken while participants watched a relaxing video. Participants then completed the emotion recognition task. The emotion recognition task included 32 trials à two blocks (16 × sunglasses, 16 × glasses). Each block consisted of four targets displaying each of the four emotional facial expressions (happiness, sadness, anger, and disgust) in random order with the restriction that the same emotional expression was shown not more than twice in a row. During target presentation, facial EMG was recorded to assess mimicry. The stimuli were shown full-screen and the radius of the glasses was 1.8 cm. After the emotion recognition task, participants rated their perceived interpersonal closeness to the target. Following this, participants completed the manipulation check. Finally, participants were fully debriefed and all outstanding questions were answered by the experimenter.

### Measures and instruments

#### Emotion recognition

Participants rated the emotional expressions of the targets on each of the following 7-point scales anchored with 1 = *not at all* and 7 = *very intensely*: sadness, happiness, disgust, anger, calmness, fear, and surprise while facial electromyography was measured (see below). Targets were presented for 4 s before the rating scales appeared. Responses were considered as accurate if the rating on the target emotion scale (i.e., anger for a person showing an angry expression) was higher than the ratings on the remaining scales. Accurate ratings were coded as 1 and inaccurate ones as 0.

#### Interpersonal closeness

After each trial, participants additionally rated their perceived interpersonal closeness to the targets using the *Inclusion of Other in the Self* Scale (IOS, Aron et al., 1992). To visualize interpersonal closeness, the scale uses Venn Diagrams ranging from 1 = *no overlap* to 7 = *almost complete overlap of other and self*. We used the concept of interpersonal closeness as a proxy for affiliation intents toward targets [e.g., Kastendieck et al., 2020; Mauersberger et al., in press (see footnote 2)].^1^

#### Facial electromyography (EMG)

Emotional mimicry was assessed using facial EMG at the *Corrugator Supercilii* (frown), *Orbicularis Oculi Lateralis* (wrinkles around the eyes), *Levator Labii Superioris Alaeque Nasi* (lifting the upper lip in disgust), and the *Zygomaticus Major* (lifting the corners of the mouth in a smile) sites on the left side of the face using bipolar placements of Easycap GmbH Ag/AgCl miniature surface electrodes filled with Signa gel by Parker Laboratories Inc. The skin was cleansed with lemon prep peeling and 70% alcohol, and impedances were kept below 30kΩ whenever possible. Raw EMG data were sampled using a MindWare bioamplifier with a 50 Hz notch filter at 1000 Hz. The signals were band pass filtered between 30 and 300 Hz.

#### EMG data preparation

The EMG data were offline rectified and smoothed. Within-subject z-transformed difference scores (trial – baseline) were calculated for each trial and each muscle to control for the individuals’ general expressiveness (their general level of facial activity). Happiness mimicry is indexed by increased O. Oculi and Zygomaticus M. as well as decreased Corrugator S. activity, sadness, and anger mimicry by the converse pattern (Hess et al., 2017). Disgust mimicry is indexed by increased Levator L. and decreased Corrugator S. activity (Wingenbach et al., 2020). The 40 100-ms bins were aggregated into four 1-s segments.

### Manipulation check

As a manipulation check, participants saw each of the eight targets from the emotion recognition task blocked by eye contact (glasses vs. sunglasses) once again, but with a neutral expression. Participants rated their perceived eye contact with the targets and indicated how much they liked the targets on 7-point scales anchored with 1 = *not at all* and 7 = *very much.*

## Results

### Manipulation check

#### Eye contact

First, we tested whether the eye contact manipulation (glasses vs. sunglasses) had an effect on participants’ perceived eye contact with the targets. As expected, a linear mixed model (LMM) analysis with eye contact (glasses vs. sunglasses) as predictor of eye contact rating revealed that participants reported to have significantly less perceived eye contact with targets wearing sunglasses, *M_sun_* = 2.02, *CI_95_sun_*[1.69, 2.35], than with targets wearing clear glasses, *M_gla_* = 5.14, *CI_95_gla_*[4.81, 5.47], *b_sun-gla_* = −3.12, *t* = −22.1, *p* < 0.001, *CI_95_sun-gla_*[−3.40, −2.84].

#### Liking

Then, we examined whether eye contact (glasses vs. sunglasses) had an effect on the liking of the targets. Here, the LMM analysis with eye contact (glasses vs. sunglasses) as predictor of liking revealed no significant difference in liking between targets wearing sunglasses and targets wearing clear glasses *b_sun-gla_* = −0.23, *t* = −1.95, *p* = 0.052, *CI_95_sun-gla_*[−0.47, 0.00]. Yet, there is a trend in the assumed direction—that is, targets with sunglasses were perceived as less likable than targets with clear glasses. It is very likely that this difference only did not become significant due to restrictions of power, as it is a rather small effect and we only have had eight (instead of 32) trials here.

#### Emotion recognition

**Figure 2:**
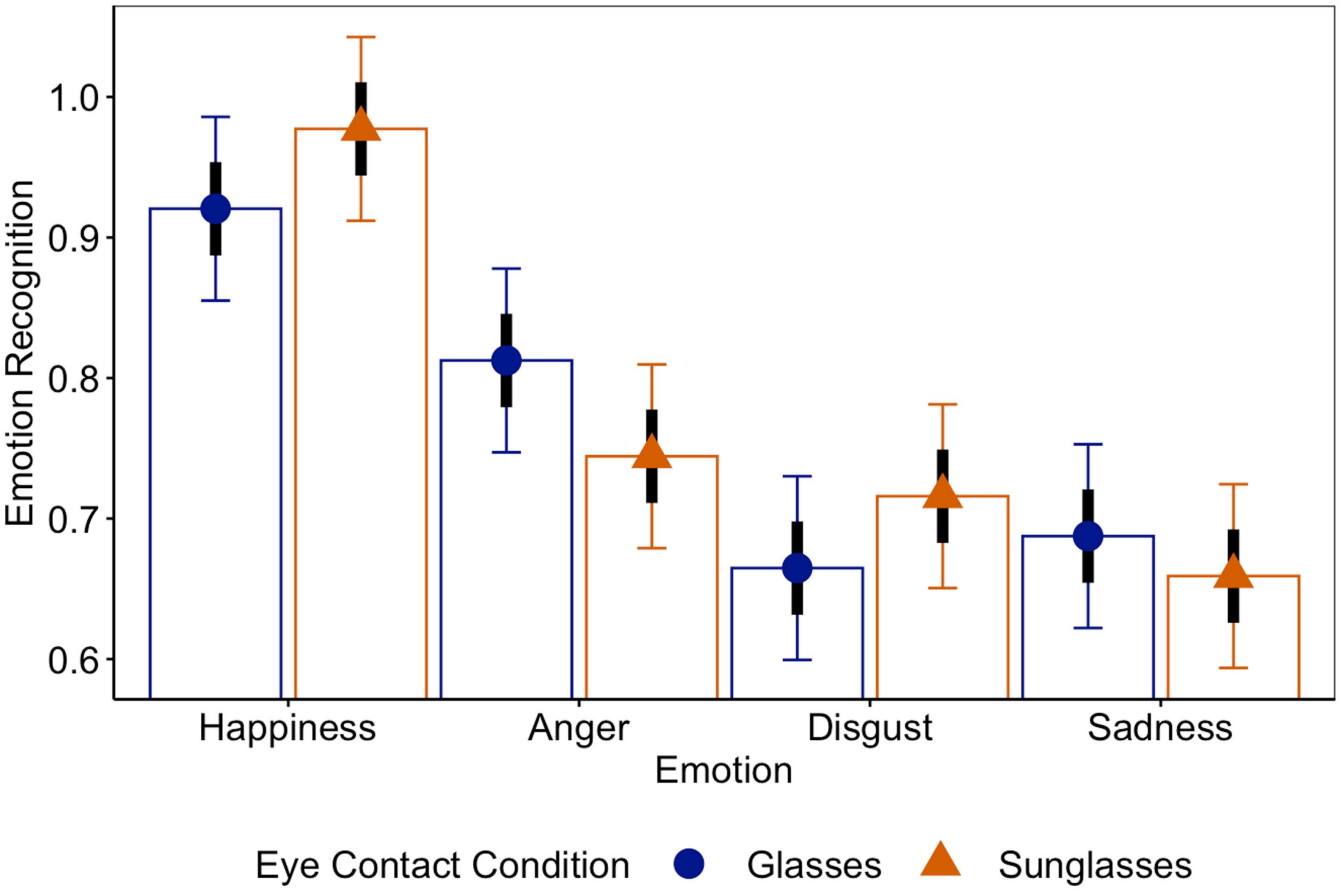
Emotion recognition as a function of eye contact and emotion.

We further tested whether eye contact (glasses vs. sunglasses) had an effect on emotion recognition accuracy. Here, the LMM analysis with eye contact (glasses vs. sunglasses) and emotion (happiness vs. anger, anger vs. disgust, and disgust vs. sadness)^2^ as predictors of emotion recognition accuracy revealed no significant difference in emotion recognition accuracy between targets wearing sunglasses and targets wearing clear glasses, *b_sun-gla_* = 0.0001, *t* = 0.14, *p* = 0.89, *CI_95_sun-gla_*[−0.04, 0.04].^3^ Yet, unsurprisingly, there was a main effect of emotion on emotion recognition accuracy: Happiness was detected best, *M_hap_ =* 95%, *CI_95_hap_*[90%, 100%], significantly better than anger, *M_ang_ =* 78%, *CI_95_ang_*[73%, 83%], *b_ang-hap_* = −17%, *t* = −5.76, *p* < 0.001, *CI_95_ang-hap_*[−23%, −11%], whereas anger was detected significantly better than disgust, *M_dis_ =* 69%, *CI_95_dis_*[64, 74%], *b_dis-ang_* = −9%, *t* = −2.89, *p* < 0.001, *CI_95_dis-ang_*[−15%, −5%], which did not significantly differ from sadness detection rates, *M_sad_ =* 67%, *CI_95_sad_*[62%, 72%], *p* = 0.56 (see Figure 2).^4^

### Hypothesis testing

#### Interpersonal closeness

**Figure 3:**
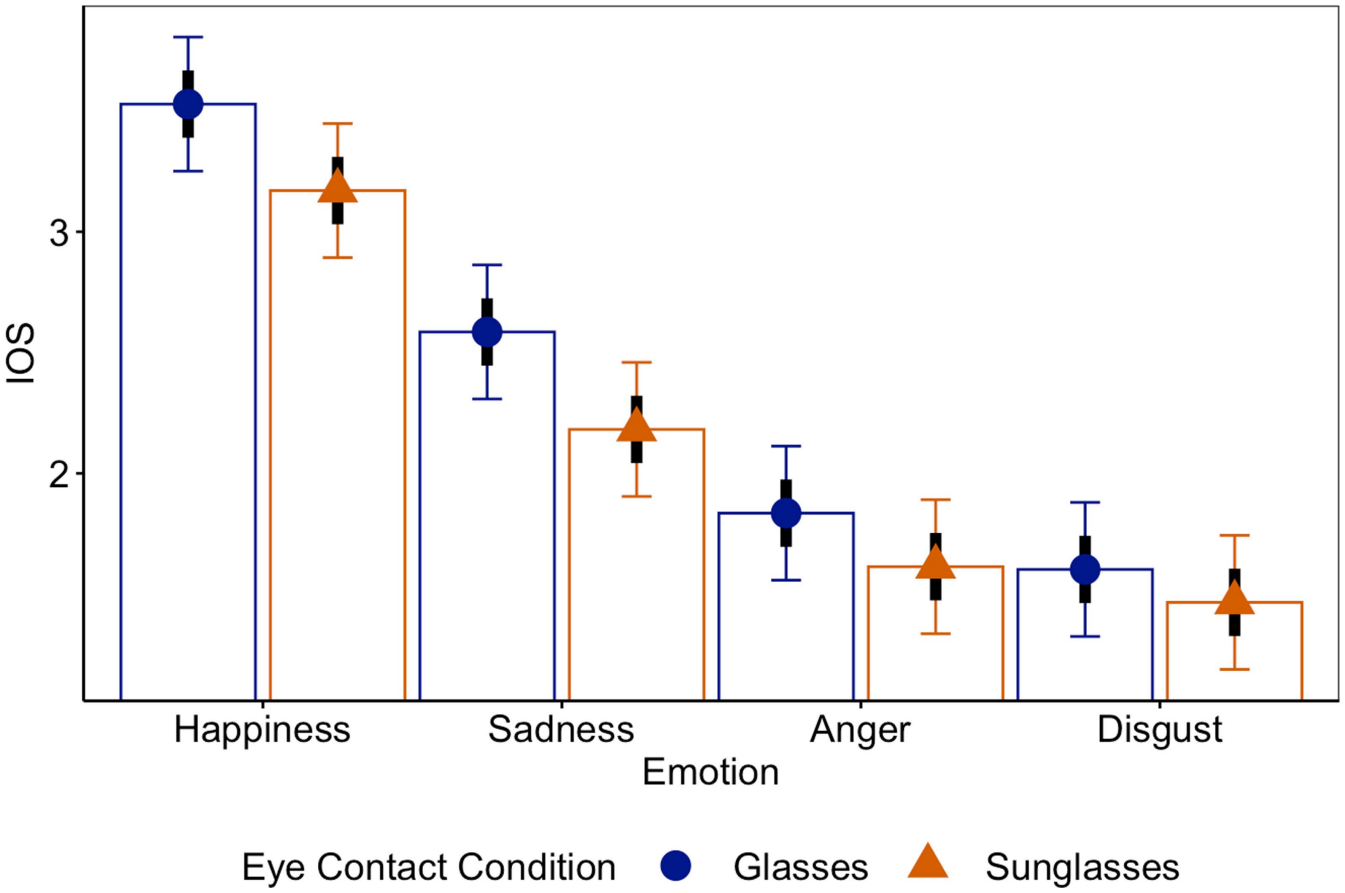
Perceived interpersonal closeness (IOS) as a function of eye contact and emotion.

We expected that eye contact (glasses vs. sunglasses) influences interpersonal closeness. Specifically, participants should feel closer to targets wearing glasses than to targets wearing sunglasses. To test this assumption, we conducted an LMM analysis with eye contact (glasses vs. sunglasses) and emotion (happiness vs. sadness, sadness vs. anger, and anger vs. disgust) as predictors of interpersonal closeness. In line with our assumption, participants reported feeling significantly less close toward targets wearing sunglasses, *M_sun_* = 2.11, *CI_95_sun_*[1.87, 2.35], compared to targets wearing clear glasses, *M_gla_* = 2.39, *CI_95_gla_*[2.15, 2.63], *b_sun-gla_* = −0.28, *t* = −4.90, *p* < 0.001, *CI_95_sun-gla_*[−0.39, −0.17]. Further, an effect of emotion on interpersonal closeness emerged: Happy targets, *M_hap_ =* 3.35, *CI_95_hap_*[3.09, 3.60], were perceived as significantly closer than sad targets, *M_sad_ =* 2.38, *CI_95_sad_*[2.13, 2.64], *b_sad-hap_* = −0.97, *t* = −11.9, *p* < 0.001, *CI_95_sad-hap_*[−1.12, −0.81], sad targets were perceived as significantly closer than angry targets, *M_ang_ =* 1.72, *CI_95_ang_*[1.47, 1.98], *b_ang-sad_* = −0.66, *t* = −8.15, *p* < 0.001, *CI_95_ang-sad_*[−0.82, −0.50], and angry targets were perceived as significantly closer than disgusted targets, *M_dis_ =* 1.53, *CI_95_dis_*[1.28, 1.79], *b_dis-ang_* = −0.19, *t* = −2.35, *p* = 0.021, *CI_95_dis-ang_*[−0.35, −0.03] (see Figure 3).

#### Emotional mimicry

We predicted that eye contact (glasses vs. sunglasses) affects emotional mimicry. Specifically, participants should mimic happiness, sadness, and anger more intensely when targets wear glasses instead of sunglasses and disgust more intensely when targets wear sunglasses instead of glasses. An LMM analysis with eye contact (glasses vs. sunglasses), emotion (happiness vs. sadness; sadness and happiness vs. anger; anger, sadness, and happiness vs. disgust), and segment as predictors of mimicry was conducted.

**Figure 4:**
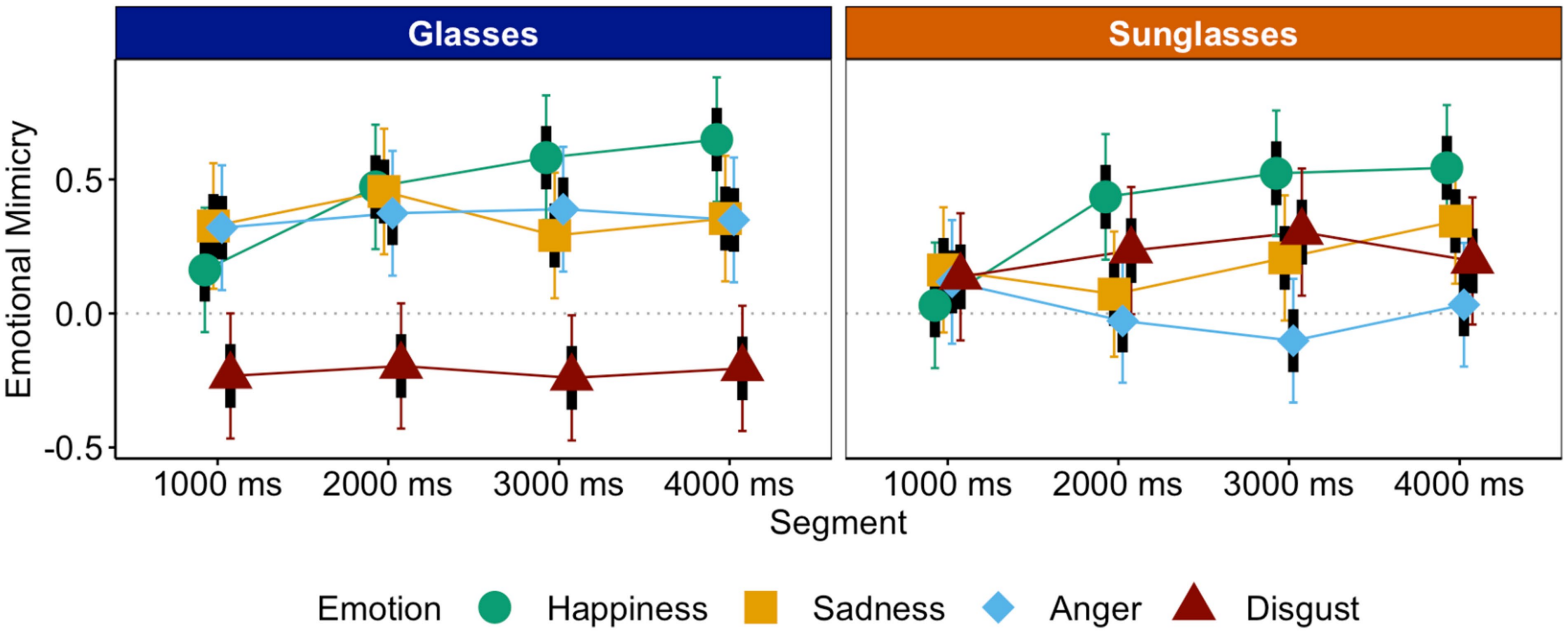
Emotional mimicry as a function of eye contact, segment, and emotion.

First, mimicry was evident across all emotions, [intercept] = 0.22, *t* = 8.15, *p* < 0.001, *CI_95_*[0.17, 0.27], even though disgust was mimicked less intensely than happiness, sadness, and anger, *b_dis-(hap,sad,ang)_* = −0.22, *t* = −6.09, *p* < 0.001, *CI_95_dis-(hap,sad,ang)_*[−0.29, −0.15]. Further, in line with predictions, disgust was mimicked more intensely in the sunglasses condition, *M_sun_dis_* = 0.22, *CI_95_sun_dis_*[0.10, 0.34], than in the glasses condition—where counter-mimicry occurred—*M_gla_dis_* = −0.22, *CI_95_gla_dis_*[−0.34, −0.10], simple slope *z_sun-gla_dis_* = 0.44, *t* = 5.18, *p* < 0.001, whereas all other emotions were mimicked more intensely in the glasses, *M_gla_(hap,sad,ang)_* = 0.39, *CI_95_gla_(hap,sad,ang)_*[0.32, 0.47], than in the sunglasses condition, *M_sun_(hap,sad,ang)_* = 0.19, *CI_95_sun_(hap,sad,ang)_*[0.12, 0.27], simple slope *z_sun-gla_(hap,sad,ang)_* = −0.20, *t* = −4.18, *p* < 0.001, b_sun-gla*dis-(hap,sad,ang)_ = 0.48, *t* = 6.57, *p* < 0.001, CI_95_sun-gla*dis-(hap,sad,ang)_[0.33, 0.62] (see Figure 4). Additionally, mimicry got stronger across segments, *b_seg_* = 0.05, *t* = 2.59, *p* = 0.009, *CI_95_seg_*[0.01, 0.08].

##### Interpersonal closeness and affiliative mimicry

Interestingly, the differences in mimicry between eye-contact conditions were less pronounced for happiness and sadness than for anger, *b_ang-(hap,sad)_* = −0.31, *t* = −4.36, *p* < 0.001, *CI_95_ang-(hap,sad)_*[−0.45, −0.17]. This may be explained by the fact that interpersonal closeness was still quite high for targets wearing sunglasses who showed affiliative emotions such as happiness or sadness (ceiling effect). Even though anger expressions elicited more interpersonal closeness than disgust expressions and were mimicked as expected for an emotion with affiliative potential, they nonetheless elicited less interpersonal closeness than happiness and sadness expressions (see Figure 2). However, the difference between conditions for anger is not larger than the difference between conditions for happiness and sadness. This suggests that reductions in interpersonal closeness reduce mimicry not incrementally but in steps. Only below a certain level of interpersonal closeness, the lack thereof has an effect in a way that mimicry may be absent or even turn into counter-mimicry. Hence, whereas happiness was mimicked in both conditions, anger was only mimicked in the more affiliative (the glasses) condition.

**Figure 5:**
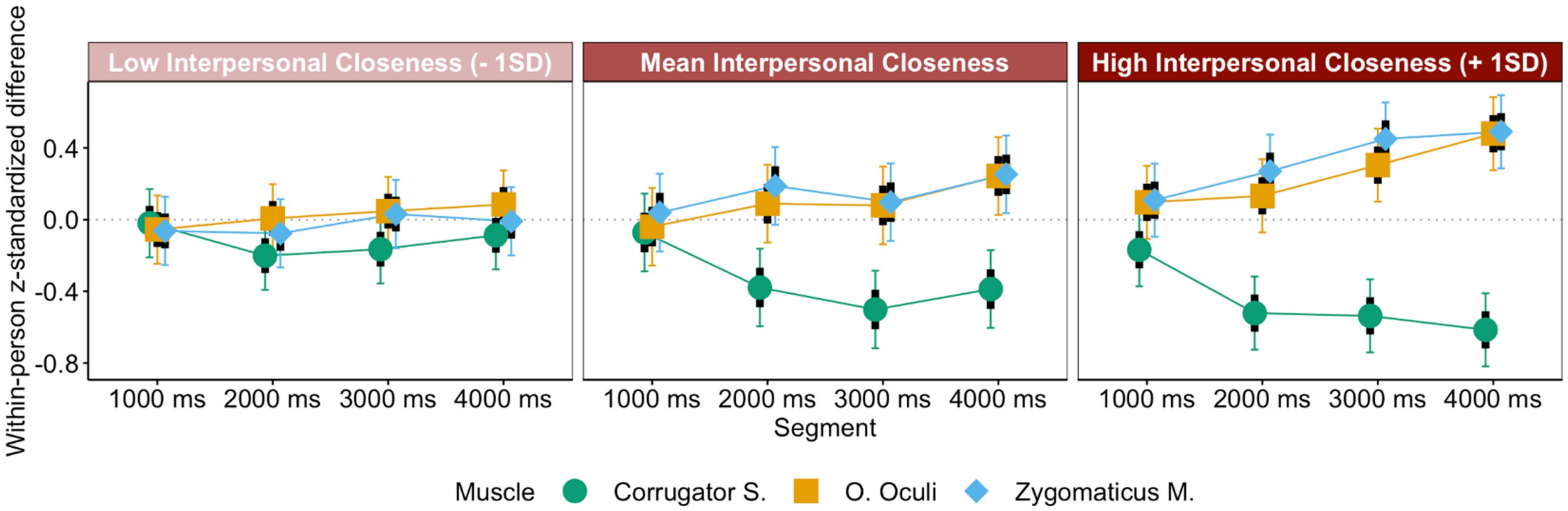
Happiness mimicry as a function of interpersonal closeness, segment, and muscle.

**Figure 6:**
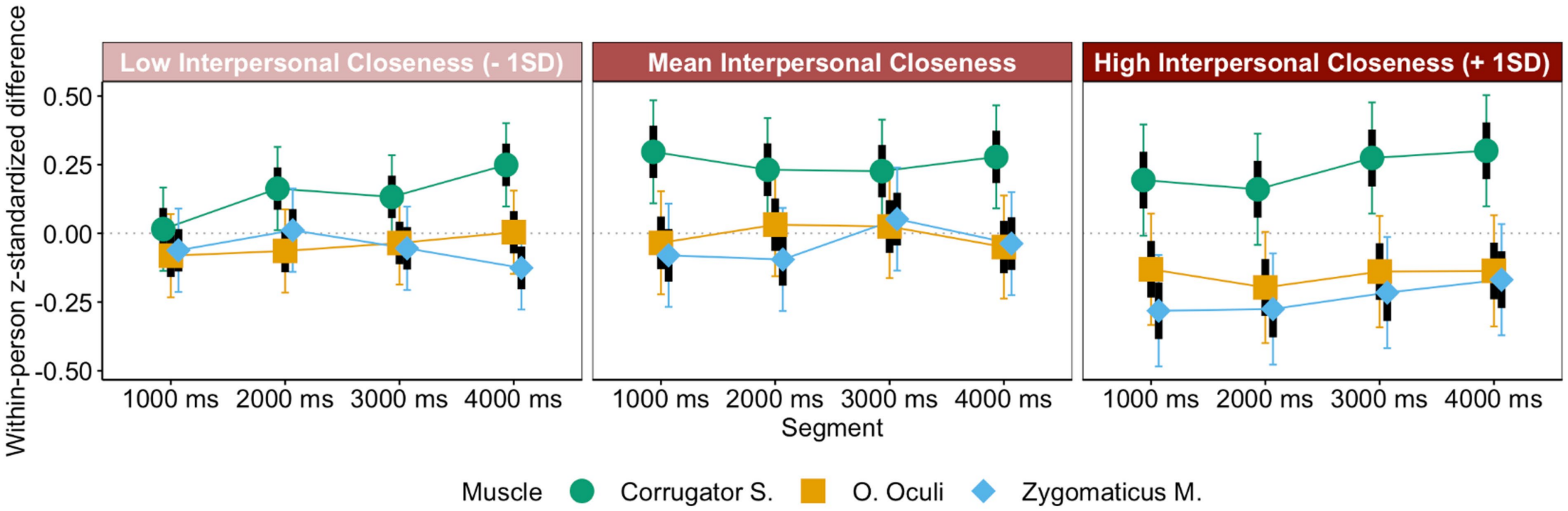
Sadness mimicry as a function of interpersonal closeness, segment, and muscle.

To follow up on these ideas, we conducted additional LMM analyses with eye contact (glasses vs. sunglasses), muscle site (Corrugator S. vs. Zygomaticus M. and O. Oculi for happiness, sadness, and anger, and Levator L. vs. Corrugator S. for disgust), segment, and interpersonal closeness as moderator on mimicry for each of the emotions separately. Interestingly, different effects emerged depending on emotion. Perceived interpersonal closeness to the target seemed to explain the effect of eye contact on happiness, b_cor-(zyg,ocl)*ios_ = −0.14, *t* = −5.35, *p* < 0.001, CI_95_cor-(zyg,ocl)*ios_[−0.19, −0.09], and sadness mimicry, b_cor-(zyg,ocl)*ios_ = 0.09, *t* = 3.21, *p* < 0.001, CI_95_cor-(zyg,ocl)*ios_[0.03, 0.14]. That is, mimicry was more pronounced when interpersonal closeness was high (+ 1 *SD*), simple slope *z_cor-(zyg,ocl)_ios-high_* = −0.61, *t* = 12.17, *p* < 0.001 (happiness), simple slope *z_cor-(zyg,ocl)_ios-high_* = 0.38, *t* = 8.52, *p* < 0.001 (sadness), than when it was low (− 1 *SD*), simple slope *z_cor-(zyg,ocl)_ios-low_* = −0.23, *t* = −4.62, *p* < 0.001 (happiness)^5^, *z_cor-(zyg,ocl)_ios-low_* = 0.18, *t* = 3.95, *p* < 0.001 (sadness; see Figures 5, 6). In fact, for happiness and sadness, the effect of eye contact was not significant once after adding interpersonal closeness as a covariate to the analyses.

**Figure 7:**
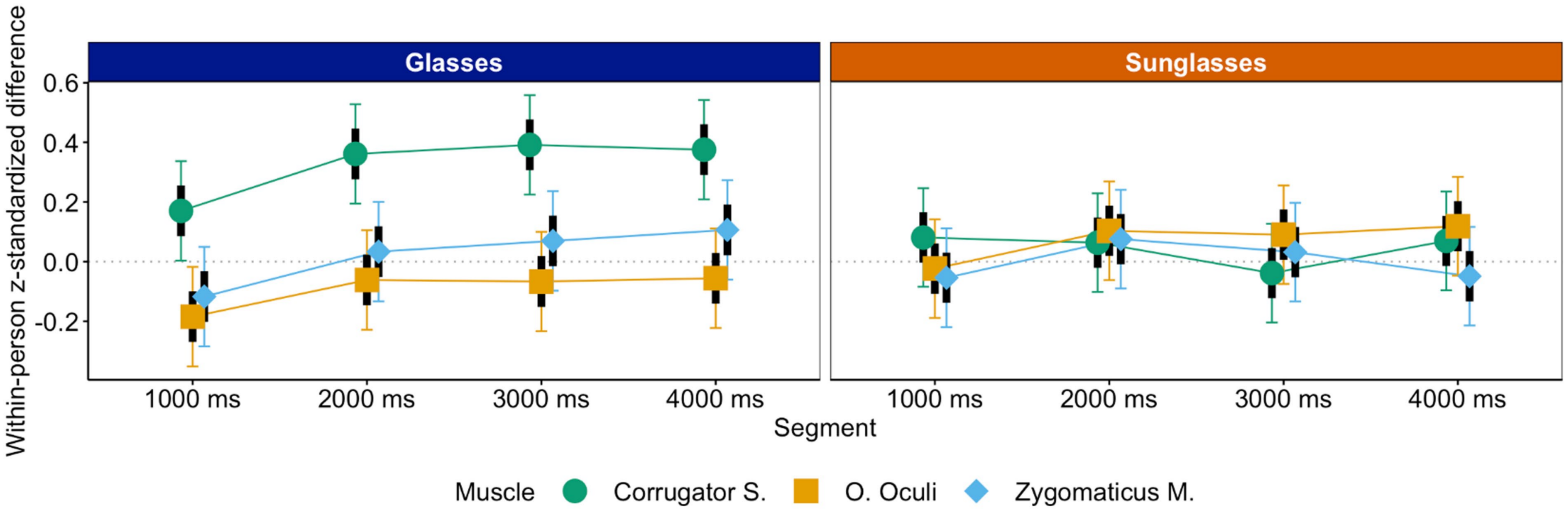
Anger mimicry as a function of eye contact, segment, and muscle.

This was not the case for anger. For anger, only eye contact explained whether or not mimicry took place, b_cor-(zyg,ocl)*sun-gla_ = −0.35, *t* = −5.01, *p* < 0.001, CI_95_cor-(zyg,ocl)*sun-gla_[−0.49, −0.21]. That is, mimicry only could be found during the glasses condition, simple slope *z_cor-(zyg,ocl)_gla_* = 0.36, *t* = 7.20, *p* < 0.001, and not during the sunglasses condition, *p* = 0.88 (see Figure 7). Hence, for anger only, wearing sunglasses had an effect on mimicry over and above what impairments in perceived interpersonal closeness could explain.^6^

**Figure 8:**
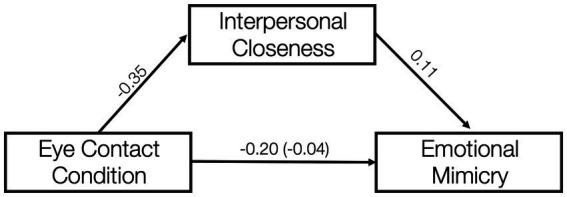
Perceived interpersonal closeness mediates the relationship between eye contact and emotional mimicry.

Nevertheless, a multilevel mediation analysis across all affiliative emotions revealed—besides the direct effect of eye contact on mimicry (−0.16, negative estimate indicates less mimicry during the sunglasses condition compared to the clear glasses condition)—an indirect effect of eye contact *via* perceived interpersonal closeness on mimicry of −0.04 (i1 = −0.35*i2 = 0.11). Hence, a substantial part of the total effect was the indirect effect (20%), suggesting that the reduction in interpersonal closeness when eye contact is obstructed is an important explanation for why obstructed eye contact impedes mimicry (see Figure 8).

##### Interpersonal closeness and disgust mimicry

**Figure 9:**
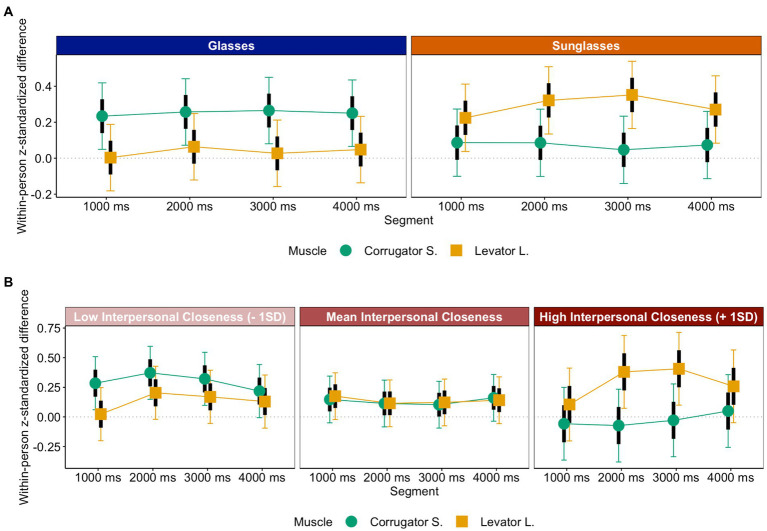
Disgust mimicry as a function of eye contact, segment and muscle **(A)** and interpersonal closeness, segment and muscle **(B)**.

For disgust, the LMM analysis with interpersonal closeness as covariate yielded effects of both eye contact, b_lev-cor*sun-gla_ = 0.47, *t* = 5.50, *p* < 0.001, CI_95_lev-cor*sun-gla_[0.30, 0.64], and perceived interpersonal closeness, b_lev-cor*ios_ = 0.20, *t* = 3.43, *p* < 0.001, CI_95_lev-cor*ios_[0.09, 0.32], however, in opposite directions. Whereas participants mimicked disgust in the sunglasses condition, simple slope *z_lev-cor_sun_* = 0.37, *t* = 4.96, *p* < 0.001, they did not mimic disgust in the glasses condition, simple slope *z_lev-cor_gla_* = −0.09, *t* = −1.36, *p* = 0.17; in contrast, participants mimicked disgust only when they felt at least a little close to the target (+ 1 *SD*), simple slope *z_lev-cor_ios_high_* = 0.15, *t* = 2.46, *p* = 0.014, and showed counter-mimicry when they felt very distant from the targets (− 1 *SD*), simple slope *z_lev-cor_ios_low_* = −0.14, *t* = −2.40, *p* = 0.016 (see Figure 9). It may be that a prior relationship or bond to the target increases the indignation and hence affective reaction if that target suddenly sneers hidden behind sunglasses. Indeed, supplementary analyses confirm that the target with the highest overall interpersonal closeness rating elicits the strongest disgust mimicry response in the sunglasses condition (see R Markdown file on OSF: http://doi.org/10.17605/OSF.IO/XMRQS).

## Discussion

Eye contact is a crucial social skill that helps to create bonds between interaction partners during communication. Famous sentences such as “Eyes are the window to the soul” (origin unknown, but often attributed to Shakespeare, Da Vinci, Cicero or even the Bible) already have acknowledged the importance of eye contact several centuries ago. Conversely, if eye contact is obstructed, the invisible thread connecting two people with each other is ripped apart. In consequence, nonverbal gestures, behaviors, and facial expressions that are important for social interaction quality such as emotional mimicry may also be affected. This can be the beginning of a vicious circle which is difficult to break. Indeed, emotional mimicry has been found to foster mutual liking and establish social connectedness and prior research confirms that black sensor bars in front of the mimickee’s eyes as well as averted gaze may impair emotional mimicry responses (e.g., Rychlowska et al., 2012; Imafuku et al., 2020; Kuang et al., 2021).

The present study used an ecologically valid approach that does not obscure the emotion expression as well by using mirrored sunglasses versus plain glasses. The results suggest that reducing eye contact with sunglasses (versus clear glasses) reduces interpersonal closeness as well as emotional mimicry. Thus, our findings highlight the relevance of eye contact for nonverbal communication tools such as emotional mimicry and hence underline the importance of eye contact for everyday life.

### The effects of eye contact and interpersonal closeness on anger mimicry

Even though both interpersonal closeness and emotional mimicry were reduced in the sunglasses compared to the glasses condition for happiness, sadness, and anger, the effect of condition was more pronounced for anger than for happiness and sadness. That is, it seems that different processes may explain the reduction in mimicry. For happiness and sadness mimicry, interpersonal closeness completely mediated the effect of sunglasses vs. clear glasses. This was not the case for anger mimicry.

Anger is not the prototype of an affiliative emotion. On the contrary, the meaning and effects of anger depend on the interplay between the person and the situation during which anger is shown (Hess, 2014; Van Doorn et al., 2014; Rothschild and Keefer, 2018; Petkanopoulou et al., 2019). It may be helpful to show anger to restore relationships in the long run but anger may also destroy momentary interaction quality and social satisfaction (Fischer and Roseman, 2007). Establishing eye contact while showing anger may signal being able to correct the perceived injustice and address interpersonal problems directly whereas hiding behind darkened spectacles while showing anger may signal a lack of engagement—maybe even rejection. That is, whereas happiness and sadness were very likely to be perceived as affiliative, whether anger was perceived as affiliative may have depended on the eye-contact condition.

In fact, precisely *because* happiness is very affiliative, it might not have been influenced that much by ambiguous cues that reduce affiliativeness only to a certain (small) extent such as wearing sunglasses (Hess, 2021; also see, Hietanen et al., 2019, for the finding that happiness mimicry does not depend on whether or not participants feel watched by their interaction partner). Indeed, individuals are biased to judge happy others whose gaze is averted as looking toward them and this is also true when eyes are invisible. This effect can be explained by the self-referential positivity bias; that is, people are more likely to associate happy others (than angry or fearful others) with the self, as it boosts one’s self-esteem to think that one has made someone else happy (Lobmaier and Perrett, 2011). Hence, across participants, targets wearing sunglasses while displaying happiness may still have been perceived as directing their gaze toward participants in most cases; hence happy targets with sunglasses generally elicited feelings of closeness that were strong enough to allow for mimicry. This may have rendered closeness a more relevant predictor of happiness mimicry than eye contact as such.

In contrast, closeness was overall low for targets displaying anger as this emotion is usually not desirable in interactions. Still, displaying anger may signal respect and competence to correct the perceived wrong (Hess, 2014). Hence, one may usually display understanding and empathy toward an angry other (i.e., anger mimicry is often found, e.g., Hess and Fischer, 2013). Yet this is only true, if individuals *do not* hide behind sunglasses. If they *do* hide behind sunglasses, anger may become maladaptive as sunglasses may counteract the feeling that the angry person is really willing to address interpersonal problems directly. Hence, the two conditions (clear glasses versus sunglasses) may represent adaptive anger versus maladaptive anger. In this sense, only adaptive anger is mimicked—but not only because participants feel closer to targets showing adaptive compared to maladaptive anger but mainly because expressing adaptive anger can be an empathic affiliative act (Van Doorn et al., 2014; Bringle et al., 2018), whereas expressing maladaptive anger may be an attempt to signal dominance in order to protect one’s ego (Fast and Chen, 2009). Dominance, in turn, does not invite the sharing of emotions but a contrasting submissive response (e.g., Hess et al., 2016).

### The effects of eye contact and interpersonal closeness on disgust mimicry

For disgust, we found a different pattern of results: Even though (similar to the effects for the other emotions) interpersonal closeness was lower during the sunglasses condition compared to the glasses condition, participants mimicked disgust only in the sunglasses condition. This is in line with our expectations: In contrast to the ambiguous nature of anger, it is an undisputed fact that disgust is an antagonistic emotion that is shown if the relationship does not have any future anymore. Disgust signals rejection and gives rise to the desire to distance from the object or subject that elicits disgust (Haidt et al., 1997; Rozin et al., 2008). Yet, if barriers such as sunglasses are situated between you and the disgusted other, it may feel easier to regard this reaction from a certain distance than if this is not the case. Hence, for antagonistic emotions such as disgust, sunglasses may be protective against the negative effects of rejection and may allow an empathic response such as mimicry to take place.

Yet, it is generally not advisable to mimic disgust, as it has negative interpersonal consequences (Mauersberger et al., 2015)—hence disgust mimicry often cannot be found at the group level of analysis (Hess and Fischer, 2013). Thus, it is actually more likely that disgust mimicry is no empathic act and hence no mimicry in the classical sense but rather an *affective* reaction that signals feelings of hurt and the lack of understanding why someone excludes and rejects one (Downey et al., 1998). Indeed, perceiving disgusted facial displays activates the same brain areas as reading descriptions of disgusting scenes or viewing disgusting pictures (Phan et al., 2002; Moll et al., 2005). In this line of reasoning, sunglasses would represent no barriers or protective shields. On the contrary, as already mentioned above, wearing sunglasses may rather signal cowardice and incompetence. It indicates not being strong enough to confront someone with a problem but hiding behind others—a very dislikable gesture. Hence, because one feels even more rejected if the other hides behind sunglasses, it may elicit a stronger affective reaction. In contrast, when someone looks at you directly, they seem open and transparent and this may be more likely to elicit irritation as a sign of standing up against it and refusing the rejection. Indeed, the pattern of muscle activity in the glasses condition not only speaks for reduced mimicry but rather for counter-mimicry, as it reverses (Corrugator S. is higher than Levator L.).

Interestingly, however, for disgust also, there was a positive effect of interpersonal closeness on mimicry. This effect seems counterintuitive at first sight, as disgust mimicry is an antagonistic act and hence far off from a positive non-verbal act that smoothens social interactions and brings individuals closer together (see above, Mauersberger et al., 2015). Hence, it should more likely take place in distant relationships or with individuals with whom one does not feel connected to such as when others wear sunglasses instead of clear glasses (which is supported by our data). Nevertheless, the difference between the two conditions was more pronounced when looking at targets to whom participants felt at least a little close. Specifically, targets that were perceived as closest to participants elicited the most intense disgust mimicry response when they hid behind sunglasses while showing disgust—probably as rejections were perceived as most humiliating when shown by likable others.

### Strengths and limitations

The present study contributes to a better understanding of the effects of eye contact for interpersonal closeness and emotional mimicry. By using a novel, more ecological valid methodology to manipulate eye contact (small round sunglasses that cover only the eye region compared to clear glasses of the same shape), we could rule out a range of alternative explanations that may explain the effects of eye contact on mimicry in previous studies.

Nevertheless, our study also some limitations. Even though we used a naturalistic stimulus set, the targets were placed in a context-free setting. Recent work by Hess and colleagues (e.g., Hess et al., 2020) suggests that the interpretation of an emotional expression is significantly influenced by the perception of the context it occurs in, and vice versa. Drawing information from the context can shape emotion perception, as it helps to give meaning to the observed expression. Further, context cues (in our study it could have been the presence of sunshine that makes wearing sunglasses plausible) may influence whether an expression is perceived as appropriate and hence whether or not mimicry takes place (Kastendieck et al., 2020; Mauersberger et al., in press (see footnote 2)). Our stimulus set comprised facial expressions shown in front of a gray background, hence lacking real-life context or interaction. Yet, during the debriefing, only one participant wondered why targets in the sunglasses condition indeed wore *sun*glasses and the results did not change excluding this participant. Further, mimicry research typically shows emotional expressions in front of a gray background and only a few recent studies included background scenes to add contextual cues (Kastendieck et al., 2020, 2021). Nevertheless, it may be interesting to explore whether the effects of clear glasses vs. sunglasses on mimicry would occur in the same manner (or possibly would be even somewhat stronger) when targets were placed in a scene (for instance, a park where the sun shines through trees in the distance).

Further, recent studies suggest that dynamic facial stimuli may allow for greater emotional engagement of observers. This raises the question of whether greater emotional engagement through dynamic emotional displays might also have affected emotional mimicry in our study, for example by enhancing the possibility for affiliation and interpersonal closeness. Indeed, an inspection of the means of interpersonal closeness suggests that overall participants felt less close to targets than the midpoint of the scale (4). Future studies could aim for higher overall perceived interpersonal closeness and thus more intense overall mimicry reactions by showing naturalistic dynamic emotional expressions instead of static stimuli (Seibt et al., 2015; Kastendieck et al., 2020, 2021; Mauersberger et al., in press (see footnote 2)).

In sum, eye contact is a powerful social cue that affects a broad range of our social interactions. Eye contact expresses attention and attitudes, dominance, and affiliation. It is a fundamental process in the development of social skills and may even have played a role in the evolution of non-human cognition and social behaviors of our ancestors (Emery, 2000; Mulholland et al., 2020). Humans as well as non-human primates behave (and feel) differently depending on whether or not they engage in eye contact and whether or not they believe their interaction partners look at them. The quality of a social interaction suffers from obstructed eye contact. One may perceive others as less likable and show less affection and empathy toward these disliked others. This may then have an impact on a broad range of subsequent social processes, which in the end then may also influence physical and psychological health (Tomasello, 2014). Hence, despite its limitations, our study poses a starting point for the aim to understand *why* eye contact is relevant for everyday social life. It affects whether or not nonverbal communication tools that signal empathy and thus smoothen social interactions such as emotional mimicry are present or absent.

## Conclusion

Establishing eye contact is an important facet of successful communication. Eye contact makes the receiver of your gaze feel closer to you, and mimic you more, and hence it helps to form relationships with others. Still, in many instances, eye contact is obstructed. Either because situations do not allow making eye contact or because we are not skilled enough to react appropriately. The results of our study suggest that these nonverbal constraints may indeed harm our relationships with others. Everybody should be aware of the risks that obstructed eye contact may bear for the satisfaction of social interaction partners and thus for social interaction quality as such.

## Data availability statement

The datasets presented in this study can be found in online repositories. The names of the repository/repositories and accession number(s) can be found at: http://doi.org/10.17605/OSF.IO/XMRQS.

## Ethics statement

The study that involved human participants was reviewed and approved by the Ethikkommission des Instituts für Psychologie der Humboldt-Universität zu Berlin. The patients/participants provided their written informed consent to participate in this study.

## Author contributions

UH had the original idea to conduct a mimicry study where obstructed eye contact was manipulated with sunglasses. UH provided the stimulus materials for the study and other resources (e.g., money) to be able to conduct the study. All authors conceptualized and designed the study in detail. HM programmed the study. HM and TK organized and supervised the data collection. HM prepared the data for analysis, performed the statistical analysis, and wrote the first draft of the manuscript. All authors contributed to manuscript revision, read, and approved the submitted version.

## Conflict of interest

The authors declare that the research was conducted in the absence of any commercial or financial relationships that could be construed as a potential conflict of interest.

## Publisher’s note

All claims expressed in this article are solely those of the authors and do not necessarily represent those of their affiliated organizations, or those of the publisher, the editors and the reviewers. Any product that may be evaluated in this article, or claim that may be made by its manufacturer, is not guaranteed or endorsed by the publisher.

## Footnotes

^1^Anger has been traditionally thought of as a negative antagonistic emotion. Yet, as anger plays an important role for the satisfaction in long-term relationships (e.g., it provides energy to correct injustice and strength to solve interpersonal problems in the long run), it may also foster bonding and affiliation (Graham et al., 2008; Hess, 2014). As we did not have a specific hypothesis with regard to how the perception of angry targets with sunglasses may differ from the perception of happy or sad targets with sunglasses, anger was treated similar to happiness and sadness in our analyses.

^1^Mauersberger, H., Kastendieck, T., Hetmann, A., Schöll, A., and Hess, U. (in press). The Phil Trans B issue on ‘Cracking the laugh code: laughter through the lens of biology, psychology and neuroscience’. Available at: https://royalsocietypublishing.org/toc/rstb/377/1863 (Accessed September 21).

^2^When an experimental factor (such as our emotion factor) has more than two levels, the fixed effect omnibus F-test is not informative with regards to where the differences exactly are. Hence, it is advised to use *a priori* contrasts to test specific differences between factor levels (Schad et al., 2020). We used repeated contrasts here to be able to "successively test neighboring factor levels against each other" (i.e., to test whether the smallest differences between factor levels are significant; Schad et al., 2020, p. 3). Prior to entering the contrasts into each analysis, the order of the factor levels had to be adjusted to the corresponding outcome variable. For instance, individuals feel closer to others who display an affiliative negative emotion such as sadness than (potentially) non-affiliative negative emotions such as anger and disgust. Still, individuals are often less accurate in recognizing sadness than anger and disgust. Hence, for emotion recognition, sadness was the lowest factor level whereas this was disgust for interpersonal closeness.

^3^Yet, note that there was an effect of eye contact (glasses vs. sunglasses) for intensity ratings. Thus, even though participants were not less accurate for targets wearing sunglasses than for those wearing clear glasses, they perceived the displayed emotions to be less intense for targets wearing sunglasses than for those wearing clear glasses, *b_sun-gla_* = −0.22, *t* = −3.17, *p* < 0.001, *CI_95_sun-gla_*[−0.36, −0.09]. An inspection of the means suggests that this difference between conditions is mainly driven by a difference for targets displaying sadness and anger (see R Markdown file on OSF).

^4^The interaction between eye contact and the difference between happiness and anger, *b_sun-gla*ang-hap_* = −12%, *t* = −2.11, *p* = 0.029, *CI_95_sun-gla*ang-hap_*[−24%, −1%], as well as the interaction between eye contact and the difference between anger and disgust, *b_sun-gla*dis-ang_* = 12%, *t* = 2.02, *p* = 0.038, *CI_95_sun-gla*dis-ang_*[0%, 24%], only indicates that there is a larger difference between the emotion recognition accuracy of happiness and anger in the sunglasses condition, simple slope *z_sun_ang-hap_* = −23%, *t* = −5.57, *p* < 0.001, than in the glasses condition, simple slope *z_gla_ang-hap_* = −11%, *t* = −2.58, *p* = 0.011, and a larger difference between the emotion recognition accuracy of anger and disgust in the glasses condition, simple slope *z_gla_dis-ang_* = −15%, *t* = −3.53, *p* < 0.001, than in the sunglasses condition, simple slope *z_sun_dis-ang_* = −3%, *t* = −0.68, *p* = 0.50. Yet, neither within happiness, nor anger, nor disgust, there was an effect of eye contact (all *p* > 0.10).

^5^For happiness, the difference in mimicry between low and high interpersonal closeness further increased with time (segments), *b_cor-(zyg,ocl)*ios*seg_* = −0.07, *t* = −2.88, *p* < 0.001, *CI_95_cor-(zyg,ocl)*ios*seg_*[−0.11, −0.02].

^6^Please note that we have added figures with eye contact as moderator for happiness and sadness and with interpersonal closeness as moderator for anger to the Supplementary Figures A–C.
