# Supplementary material for: I looked at you, you looked at me, I smiled at you, you smiled at me—The impact of eye contact on emotional mimicry
Source: Front Psychol. 2022 Sep 29;13:970954. doi: 10.3389/fpsyg.2022.970954 (PMC9556997; doi:10.3389/fpsyg.2022.970954)
Supplement: Supplementary file 1 [file Data_Sheet_1.docx]

Supplementary Materials to “I looked at you, you looked at me, I smiled at you, you smiled at me—The impact of eye contact on emotional mimicry”

Heidi Mauersberger*, Till Kastendieck, Ursula Hess

Department of Psychology, Humboldt-Universität zu Berlin, Berlin, Germany

*** Correspondence:**Heidi Mauersberger
heidi.mauersberger@hu-berlin.de

# Figures

Figure A
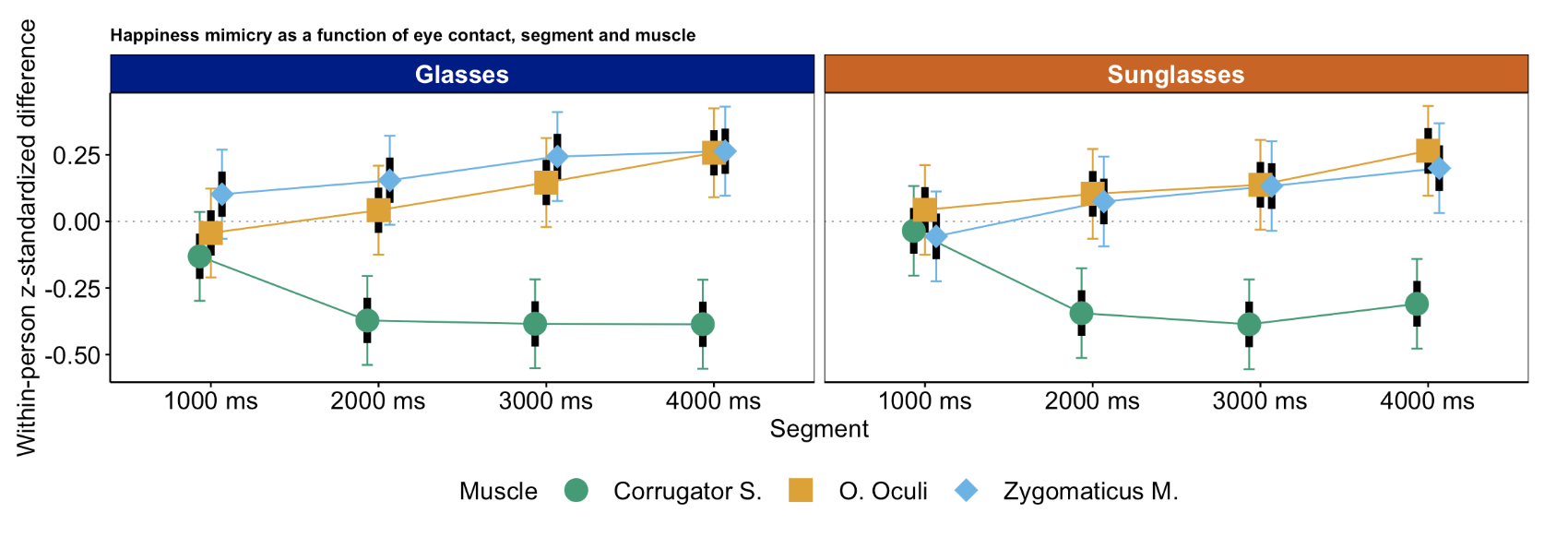


Figure B


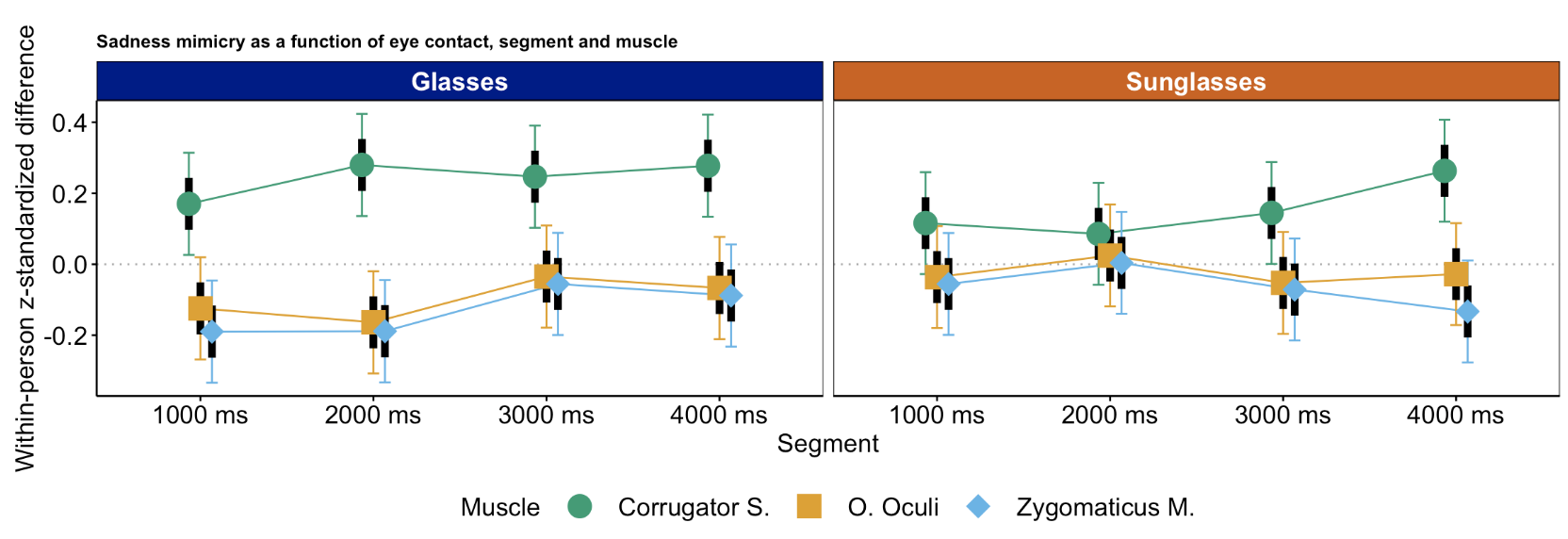


Figure C


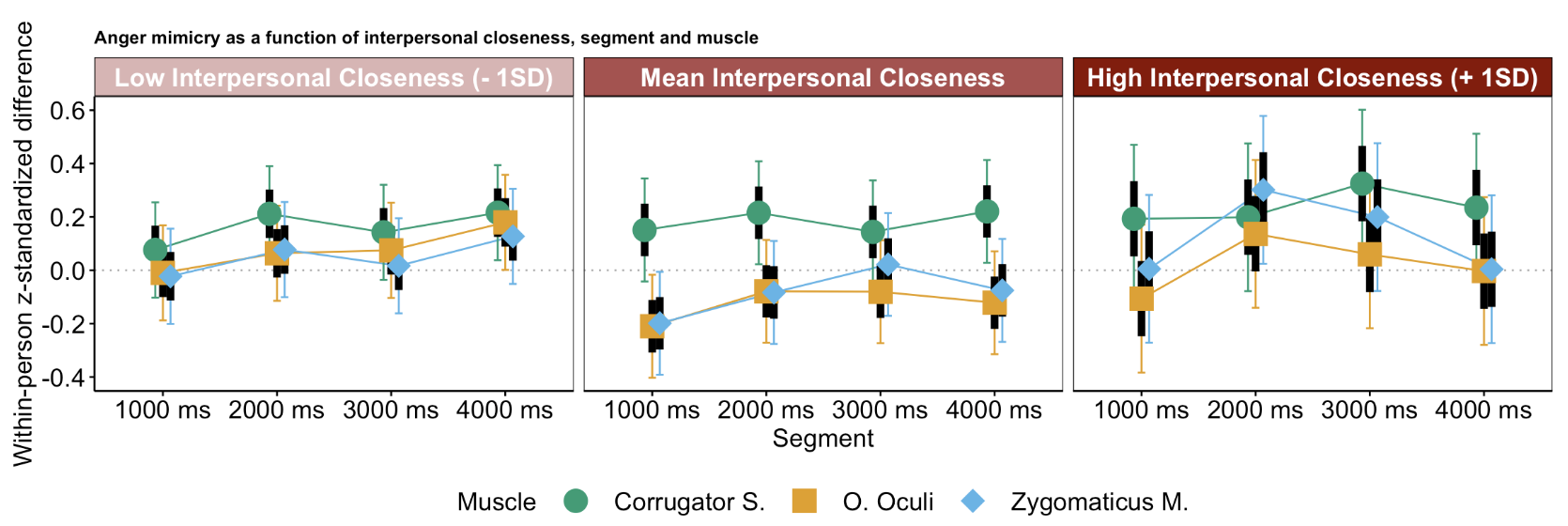


## Figure captions

*Figure A.* Happiness mimicry as a function of eye contact, segment and muscle.

*Figure B.* Sadness mimicry as a function of eye contact, segment and muscle.

*Figure C.* Anger mimicry as a function of interpersonal closeness, segment and muscle.
